# A Scheduling Algorithm for Cloud Computing System Based on the Driver of Dynamic Essential Path

**DOI:** 10.1371/journal.pone.0159932

**Published:** 2016-08-04

**Authors:** Zhiqiang Xie, Xia Shao, Yu Xin

**Affiliations:** College of Computer and Technology, Harbin University of Science and Technology, Harbin, Heilongjiang, China; Chongqing University, CHINA

## Abstract

To solve the problem of task scheduling in the cloud computing system, this paper proposes a scheduling algorithm for cloud computing based on the driver of dynamic essential path (DDEP). This algorithm applies a predecessor-task layer priority strategy to solve the problem of constraint relations among task nodes. The strategy assigns different priority values to every task node based on the scheduling order of task node as affected by the constraint relations among task nodes, and the task node list is generated by the different priority value. To address the scheduling order problem in which task nodes have the same priority value, the dynamic essential long path strategy is proposed. This strategy computes the dynamic essential path of the pre-scheduling task nodes based on the actual computation cost and communication cost of task node in the scheduling process. The task node that has the longest dynamic essential path is scheduled first as the completion time of task graph is indirectly influenced by the finishing time of task nodes in the longest dynamic essential path. Finally, we demonstrate the proposed algorithm via simulation experiments using Matlab tools. The experimental results indicate that the proposed algorithm can effectively reduce the task Makespan in most cases and meet a high quality performance objective.

## Introduction

Due to the high-speed development and popularization of the internet, network resource sharing has emerged; cloud computing is a service related to network resources, and in recent years, cloud computing has become well developed. Because cloud computing exists in a multiple network environment, its resources and services have many features, such as diversification, dynamic behaviour, and pay-per-use, among others. Different resources and services are allocated to meet different user needs. Under these conditions, cloud computing is applied in many different fields, including the finance, manufacturing, medicine, and the electronic industry, etc. Cloud computing is also represents further development of parallel computing, distributed computation and grid computing [1–6], and therefore, it is necessary to study cloud computing scheduling algorithms.

The cloud computing system creates a shared network resource and service with the user. Due to its diversified, dynamic and flexible nature, different resources and services are offered to different users, which is an advantage of cloud computing. These natures pose a new challenge for development of scheduling algorithms in the cloud computing system [7–9].

The task-scheduling algorithm a branch of scheduling algorithm, which is used to study dependent or independent tasks in a homogeneous or heterogeneous environment. To solve the task-scheduling problem, some researchers have proposed many effective and feasible scheduling algorithms, which can be categorized into list-scheduling algorithms and scheduling algorithms based on task duplication, clustering and random search. According to the scheduling system features, the scheduling algorithms are divided into two classes, namely, scheduling algorithms for homogeneous or heterogeneous environments. The main concept of the list-scheduling algorithm is that the sort order list of task nodes is obtained from the rank value of all task nodes, and the algorithms select task node from the order list in sequence for the corresponding server. The scheduling result is obtained when all task nodes are completed. The classical list-scheduling algorithm includes DLS (dynamic level scheduling) [10],HPRV&HURV [11]etc. Most list-scheduling algorithms solve the independent task scheduling problem based on a homogeneous environment, which make the algorithms simple and feasible with low time complexity. The main concept of scheduling algorithm based on task duplication is to redundantly copy task node with task graph, which reduces the communication cost between task nodes. The algorithm DVFS [12],EAD&PEBD [13],DSH [14]are proposed based on task duplication. Most scheduling algorithms based on task duplication are designed to solve the independent task scheduling problem based on a homogeneous environment. The main concept of scheduling algorithm based on clustering is to map all task nodes to the clusters. If certain task nodes are allocated to the same cluster, i.e., the task nodes are processed on the same server, then these task nodes are scheduled on the same server using the order list, such as FCBWTS [15],MCAR [16]. Most scheduling algorithms based on clustering are proposed based on a heterogeneous environment. The main concept of scheduling algorithm based on random search is to search the solution space for the scheduling problem using randomly oriented selection, such as the genetic algorithm [17], a genetic-simulated annealing algorithm [18], which often have high time complexities. The optimal solution control variable of task graph A is obtained by the scheduling algorithm, which is not suitable for finding the optimal solution of task graph B. Therefore, the scheduling algorithms might not be able to find an optimal solution control variable for most task graphs. The previous scheduling algorithms are designed to solve either the scheduling problem for independent tasks in a heterogeneous environment or the scheduling problem for dependent tasks in a homogeneous environment. The cloud computing system is a heterogeneous environment, and thus, the list scheduling algorithms are not sufficiently capable of solving the scheduling problem for dependent tasks in the heterogeneous environment, that is the task-scheduling problem appears in the cloud computing system.

In recent years, certain researchers have proposed many dependent task scheduling algorithms for heterogeneous systems. These algorithms consider both the task node itself and the communication cost between task nodes, such as HEFT&CPOP [19],HEFT-Lookahead [20],CEFT [21], which sort all task nodes prior to the actual scheduling by the task graph itself. The list scheduling algorithms do not consider the problem of scheduling order change of task nodes in the scheduling process, which is assessed by the actual computation time (cost) and communication time (cost) of task node in the scheduling process.

The main contributions of this paper are summarized as follows: we consider first the problem of scheduling order for all task nodes affected by the actual computation time (cost) and communication time (cost) of task node in the scheduling processing. To solve the problem, this paper proposes a scheduling algorithm for the cloud computing system based on the driver of dynamic essential path (DDEP). According to the constraint relations among task nodes in the scheduling model, the algorithm proposes the predecessor-task layer priority strategy to solve the scheduling order relation among task nodes problem. The dynamic essential long path strategy is proposed to compute the dynamic essential path of the pre-scheduling task nodes based on the actual computation cost and communication cost of task nodes in the scheduling process. The task node that has the longest dynamic essential path is scheduled first.The experimental results show that the proposed algorithm can effectively reduce the task Makespan in most cases and also meet a high quality performance objective.

The rest of the paper is organized as follows: section 2 reviews some related work and section 3 gives the scheduling model. Section 4 presents the proposed scheduling algorithm, and section 5 illustrates the example analysis. Its time complexity and the experimental result are presented in sections 6 and 7, respectively. Finally, some concluding remarks are given in section 8.

## Related Work

Those algorithms that consider both the computational cost of task node itself and the communication cost between task nodes have a common feature, which is that the task-scheduling result is obtained before the task scheduling. In contrast, in the proposed algorithm, the task-scheduling result is obtained after the task scheduling is obtained because the task node sort order is dynamically modified according to the actual scheduling situation. A simple comparative analysis of the proposed algorithm and the existing scheduling algorithms is described as the following sections.

### (1) HEFT

HEFT [19](heterogeneous earliest finish time) computes the rank value for all task nodes based on the mean computational cost of the task node itself and the communication cost between task nodes. The task node order list is generated by sorting all task nodes according to decreasing order of rank value. The task nodes are selected one by one from the order list and sent to the corresponding server, which minimizes the finish time of task node. The algorithm is simple and viable, and its time complexity is *O*(*n*^2^ * *k*), where the number of task nodes is *n*; and *k* is the number of servers.

The sort order of all task nodes is obtained before task graph is scheduled by HEFT algorithm, and the sort order of task node is obtained in the task graph scheduling process by the proposed algorithm (DDEP). The sort order generated by DDEP algorithm is more reasonable than HEFT algorithm. However, the dynamic essential path for each task node is computed twice by DDEP algorithm, which adds to the time complexity compared with HEFT algorithm.

### (2) HEFT-Looahead

HEFT-Lookahead [20] algorithm is an improved version of HEFT algorithm that obtains the sort order list of all task nodes in the task graph using HEFT algorithm and selects the task nodes one by one from the order list for delivery to the corresponding server, which minimizes the current task node and its successor task node finish time.

The start time for each task node is determined by HEFT-Lookahead algorithm. The current task node and its successor task node are re-scheduled on all servers, and the server is selected that minimizes the current task node and its successor task node finish time. However, the dynamic essential path is computed once, and the start time of the group task nodes is determined by DDEP algorithm. In this way, HEFT-Lookahead algorithm has a higher time complexity than DDEP algorithm, i.e., *O*(*n*^3^), where *n* is the number of task nodes.

### (3) CEFT

The main concept of algorithm [21] divides task graph into many sub-task-graphs according to the CCPs (constrained critical paths) and schedules the sub-task graph separately. The algorithm minimizes the finish time of the single task node and also minimizes the finish time of the sub-task-graph, which includes many task nodes. Compared with DDEP algorithm, CEFT algorithm considers a broader view of the task graph, which means that CEFT algorithm displays high time complexity, i.e., *O*(*n*^3^ * *k*), where *n* is the number of task nodes, and *k* is the servers number.

### (4) DCP

DCP(dynamic critical path) [22] algorithm searches for the critical path of task graph according to the absolute earliest possible start time (AEST) and the absolute latest possible start time (ALST) of task node, re-schedules the task node on the critical path on each server, and selects the server that minimizes the current task node and its successor task node finish time. The AEST and ALST of task node are updated, the previous operations are repeated, and the scheduling result is obtained when all task nodes are completely scheduled. Because the start time of each task node is determined, its successor task node will be re-scheduled once, which gives DCP algorithm a high time complexity, i.e., *O*(*n*^3^), where *n* is the number of task nodes. Compared with DDEP algorithm, the dynamic essential path is computed once, and the start time of one group task nodes is determined by DDEP algorithm, which gives DDEP algorithm a low time complexity. Furthermore, the DDEP algorithm is simple and viable.

## Data Model

**Fig 1:**
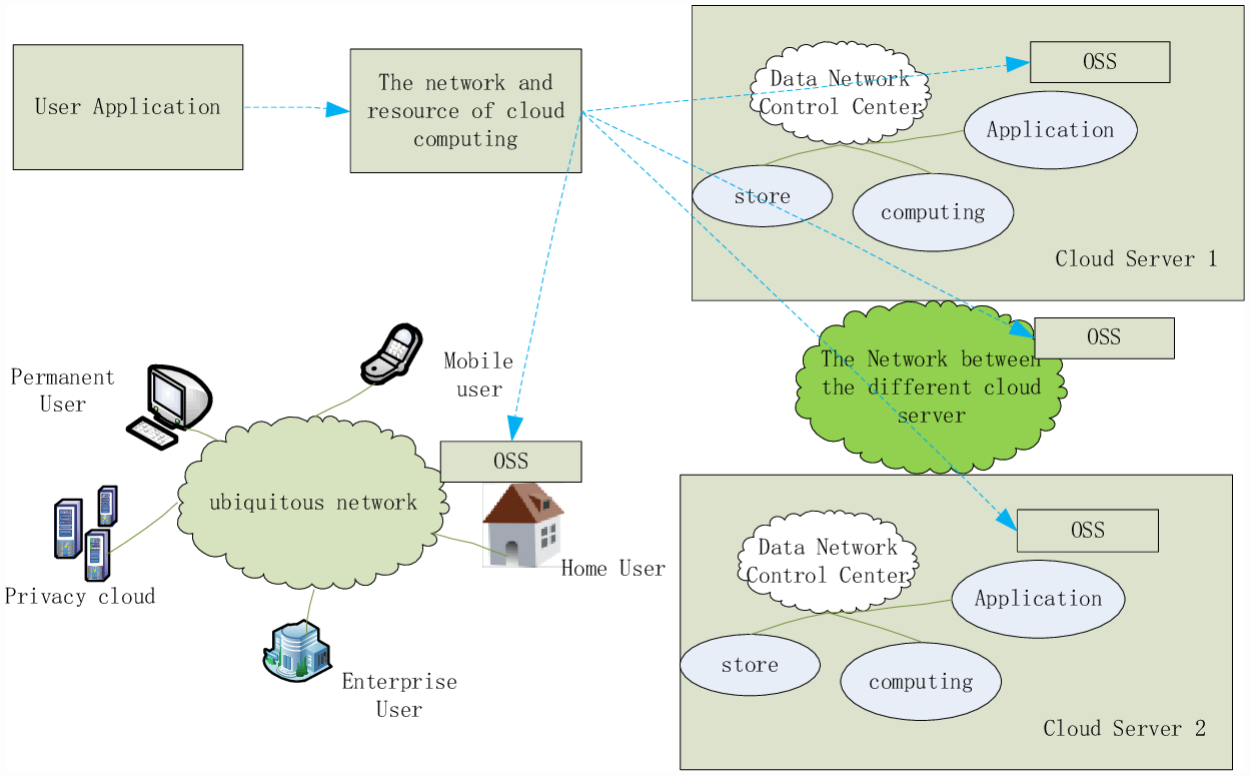
Cloud Computing Model.

The cloud computing system is a computer network composed of user applications, the network environment and an easily extensible scheduling algorithm. The users obtain the required resources and services via the scheduling algorithm in the network environment, as shown in Fig 1.

To explicitly describe the data model, we define selected terminology:

Single entry task node: If there is no parent task node referred to as a single entry task node, then its set of immediate predecessor task nodes is empty.
Single exit task node: If there are no child task node known as a single exit task node, its set of immediate successor task nodes is empty.

The main task is task node scheduling and resource allocation problem in the cloud computing system.We first create the scheduling model by converting the cloud computing scheduling problem into the DAG scheduling problem [21]. The application is simplified using the DAG graph: *G* = {*Q*,*E*,*S*}, where *Q* is the task node set of DAG graph, *Q* = {*Q*_1_,*Q*_2_,…,*Q*_*n*_}, *Q*_*i*_ represents the *ith* task node, *n* represents the number of task nodes; *S* is the set of network servers, *S* = {*S*_1_,*S*_2_,…, *S*_*m*_}, *S*_*m*_ represents the *mth* server, *m* represents the number of servers, that is the processing machine of task node; *S*_*Qi*_ is the selectable server set of *Q*_*i*_, *S*_*Qi*_⊆*S*, *T*_*Qi*_ is the computation time set of *Q*_*i*_ on the *S*_*Qi*_; *T*_*Qi*_ = {*t*_*i*__1_,*t*_*i*__2_,…,*t*_*im*_}; *t*_*im*_ represents the computation time of *ith* task node on the *mth* server. *E* is the set of communication costs among task nodes, *E* = {*e*_*ij*_}(*i*,*j*∈*Q*), and *e*_*ij*_ represents the precedence constraint relations such that *Q*_*i*_ should complete its execution before *Q*_*j*_ begins.

The DAG scheduling problem is described as follows: *EST*(*Q*_*i*_, *S*_*m*_) represents the earliest start time for *Q*_*i*_ on the server *S*_*m*_; and *EFT*(*Q*_*i*_, *S*_*m*_) represents the earliest finish time of *Q*_*i*_ on the server *S*_*m*_. For the single entry task node *Q*_*i*_ on the server *S*_*m*_:

$$EST\left(Q{}_{i},S{}_{m}\right)=T{}_{0}$$ (1)

$$EFT\left(Q{}_{i},S{}_{m}\right)=EST\left(Q{}_{i},S{}_{m}\right)+t{}_{i}{}_{m}$$ (2)

where *T*_0_ represents the application start time. For the other task nodes in the DAG graph:

$$EST\left(Q{}_{j},S{}_{m}\right)=\max_{Q_{i}\in pre\left(Q_{j}\right)}\left(EFT\left(Q{}_{i},S{}_{n}\right)+e{}_{i}{}_{j}\right)+T{}_{0}$$ (3)

$$EFT\left(Q{}_{j},S{}_{m}\right)=t{}_{j}{}_{m}+EST\left(Q{}_{j},S{}_{m}\right)$$ (4)

where *Pre*(*j*)is the set of immediate predecessor task nodes of *Q*_*j*_. After all immediate predecessor task nodes of *Q*_*j*_ are finished, the data are transmitted to *Q*_*j*_; where *e*_*ij*_ represents the communication cost between *Q*_*i*_ and *Q*_*j*_. When all data required for *Q*_*j*_ have arrived, the server *S*_*m*_ begins to process *Q*_*j*_, *e*_*ij*_ = *data*/*b*_*nm*_, where ‘data’ is the size of transmit data, and *b*_*nm*_ represents the network bandwidth from *S*_*n*_ to *S*_*m*_.

The objective functions of all task nodes on the DAG graph are described as:

$$Makespan=max(EFT\left(Q{}_{e}{}_{x}{}_{i}{}_{t}\right),S{}_{m})$$ (5)

where *Q*_*exit*_ is a single exit task node, and the final objective is to minimize the total time of the application i.e., *min*(*Makespan*).

## Scheduling Algorithm

The goal of scheduling algorithm is to minimize the completion time (execution cost) of task graph. The start time and finish time of each task node are the factors that influence the completion time of task graph, which is related to the computation time (cost) and communication time (cost). The sort order of all task nodes is an important factor that influences the start time and finish time of each task node. To solve the sort order of the task node problem, the predecessor-task layer priority strategy and the dynamic essential long path strategy are proposed. According to the sort order list, we select the server that minimizes the start time and finish time of task node.

### The Predecessor-task Layer Priority Strategy

**Fig 2:**
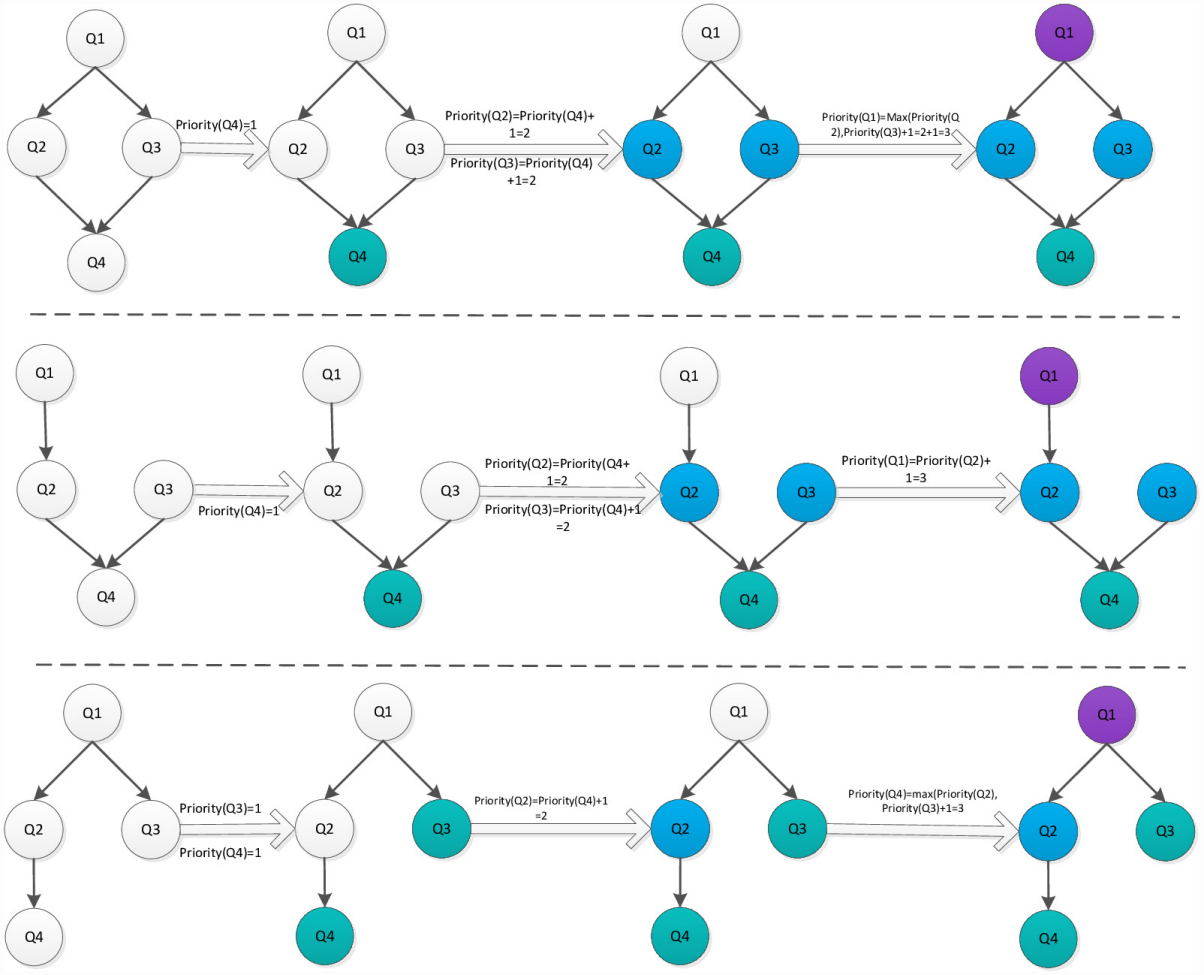
Priority value of task node with different type DAG graphs.

**Fig 3:**
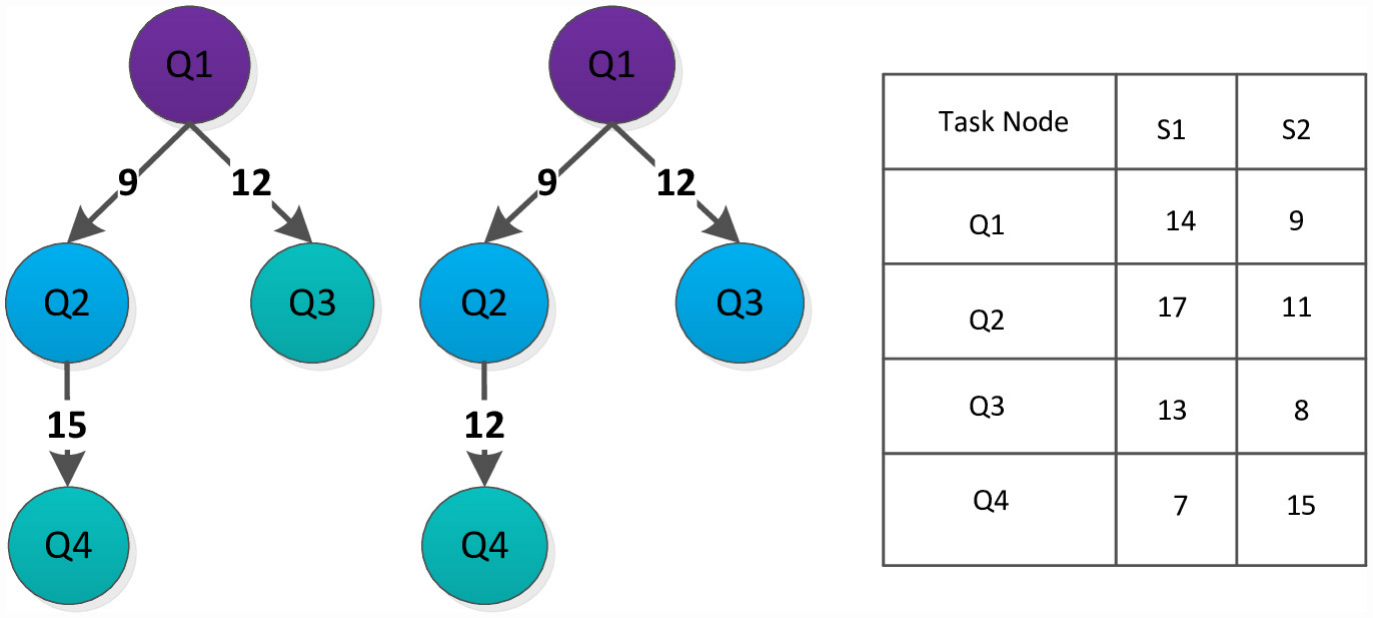
DAG graph with Non-Single-exit task node.

**Fig 4:**
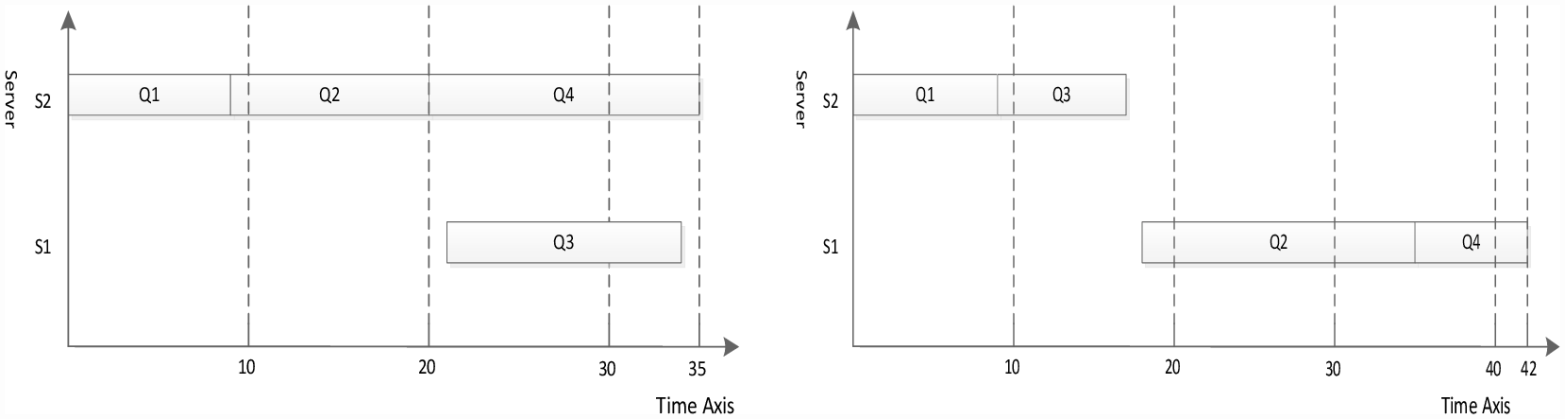
Scheduling task graph in Fig 3 using the traditional level priority (right) and the predecessor-task layer priority strategy (left).

According to the constraint relations among task nodes in the DAG model, the current task nodes are scheduled after its predecessor task nodes are finished. The predecessor-task layer priority strategy is proposed to address the scheduling order relation among task nodes problem. The entry task nodes can be first scheduled according to the scheduling order of task nodes in the task graph. When the entry task nodes are finished, the successor task nodes can be scheduled such that the entry task nodes have the top priority compared with other task nodes in the task graph. The exit task nodes have the lowest priority values, and the strategy defines the priority value of the exit task node as 1. The priority values of other task nodes in the task graph are equal to the summation of the priority values of its predecessor task node and 1. The priority values of all task nodes are obtained in turn, and the formula is defined by:

$$Priority(Q{}_{e}{}_{x}{}_{i}{}_{t})=1$$ (6)

$$Priority\left(Q{}_{j}\right)=\max_{Q_{i}\in succ\left(Q_{j}\right)}(Priority\left(Q{}_{i}\right)+1$$ (7)

where *Q*_*exit*_ is the exit task node, and *Succe*(*Q*_*j*_)is a set of the successor task nodes of *Q*_*i*_. The Fig 2 illustrates the priority value of task node in the different type task graphs. A difference exists between the predecessor-task layer priority strategy and the traditional level priority [23], in that the proposed strategy considers a broader view of task graph in which the task node obtains a higher priority value on the additional task-node path. For example, if the task graph contains multi exit task nodes, the priority values are equal for all exit task nodes in the task graph (the priority values are also 1). The task node will obtain a higher priority value for the greater number of task nodes path using the predecessor-task layer priority strategy shown in Fig 3. In other words, the task node will apply the scheduling first on the more-task-node path. In this manner, the completion time of task graph is shortened. The Fig 4 shows the scheduling result of Fig 3. According to contrast analysis of the scheduling result, the completion time of task graph with the predecessor-task layer priority strategy is better than that of the traditional level priority by 20%.

Because the priority value of Q2 is higher than that of Q3 according to the predecessor-task layer priority strategy, Q2 obtains the first scheduling choice, which shortens the completion time of task graph in Fig 3. However, the priority value of Q2 is equal to that of Q3 according to the traditional level priority strategy. If Q2 has the predecessor task node, Q3 obtains the first scheduling choice, which lengthens the completion time of the task graph in Fig 3.

### The dynamic essential long path strategy

Different priority values are assigned to all task nodes, to address the scheduling order problem for the same priority value of task nodes, the dynamic essential long path strategy is proposed. The main concept of this strategy is to first schedule the task node that has the longest essential path because the completion time of task nodes in the essentially longer path will directly influence the completion time of task graph. The dynamic essential path formula is defined by:

$$DEP\left(Q{}_{e}{}_{n}{}_{t}{}_{r}{}_{y}\right)=\bar{t{}_{e}{}_{n}{}_{t}{}_{r}{}_{y}}$$ (8)

$$DEP\left(Q{}_{j}\right)=\max_{Q_{i}\in Pre\left(Q_{j}\right)}\left(DEP\left(Q{}_{i}\right)\right)+e{}_{i}{}_{j}$$ (9)

where *Q*_*entry*_ is the entry task node, $\bar{T{}_{e}{}_{n}{}_{t}{}_{r}{}_{y}}$ is the mean computation time (cost)on all servers. *Pre*(*Q*_*j*_)is a set of the predecessor task nodes of *Q*_*i*_.

**Fig 5:**
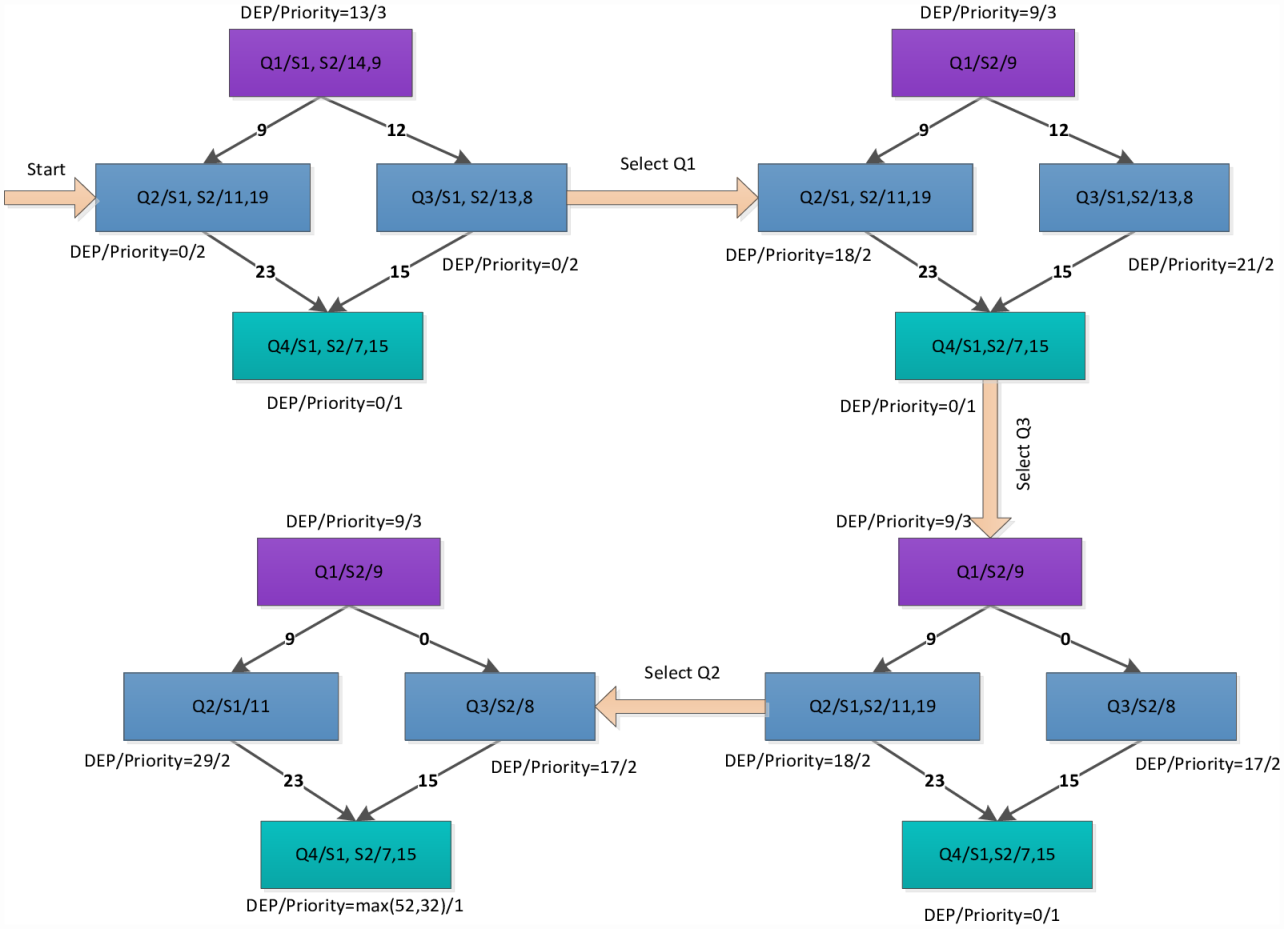
Example illustrating the procedure of the dynamic essential long path strategy.

To accurately compute the dynamic essential path of task node, the communication time (cost) is reduced to 0 by the dynamic essential long path strategy,i.e., *e*_*ij*_ = 0, when the two task nodes *Q*_*i*_ and *Q*_*j*_ are scheduled on the same server, and *Q*_*i*_ is a predecessor task node of *Q*_*j*_. When some task nodes have been finished, update their actual computation cost, server and the dynamic essential path value. This formula is defined by:

$$DEP\left(Q{}_{e}{}_{n}{}_{t}{}_{r}{}_{y}\right)=t{}_{e}{}_{n}{}_{t}{}_{r}{}_{y}{}_{k}$$ (10)

$$DEP\left(Q{}_{j}\right)=\max_{Q_{i}\in Pre\left(Q_{j}\right)}\left(DEP\left(Q{}_{i}\right)\right)+e{}_{i}{}_{j}+t{}_{j}{}_{k}$$ (11)

where *t*_*entryk*_ is the computational cost of *Q*_*entry*_ on the server *S*_*k*_. Fig 5 shows an example illustrating the procedure of the dynamic essential long path strategy.

## Example Analysis

**Fig 6:**
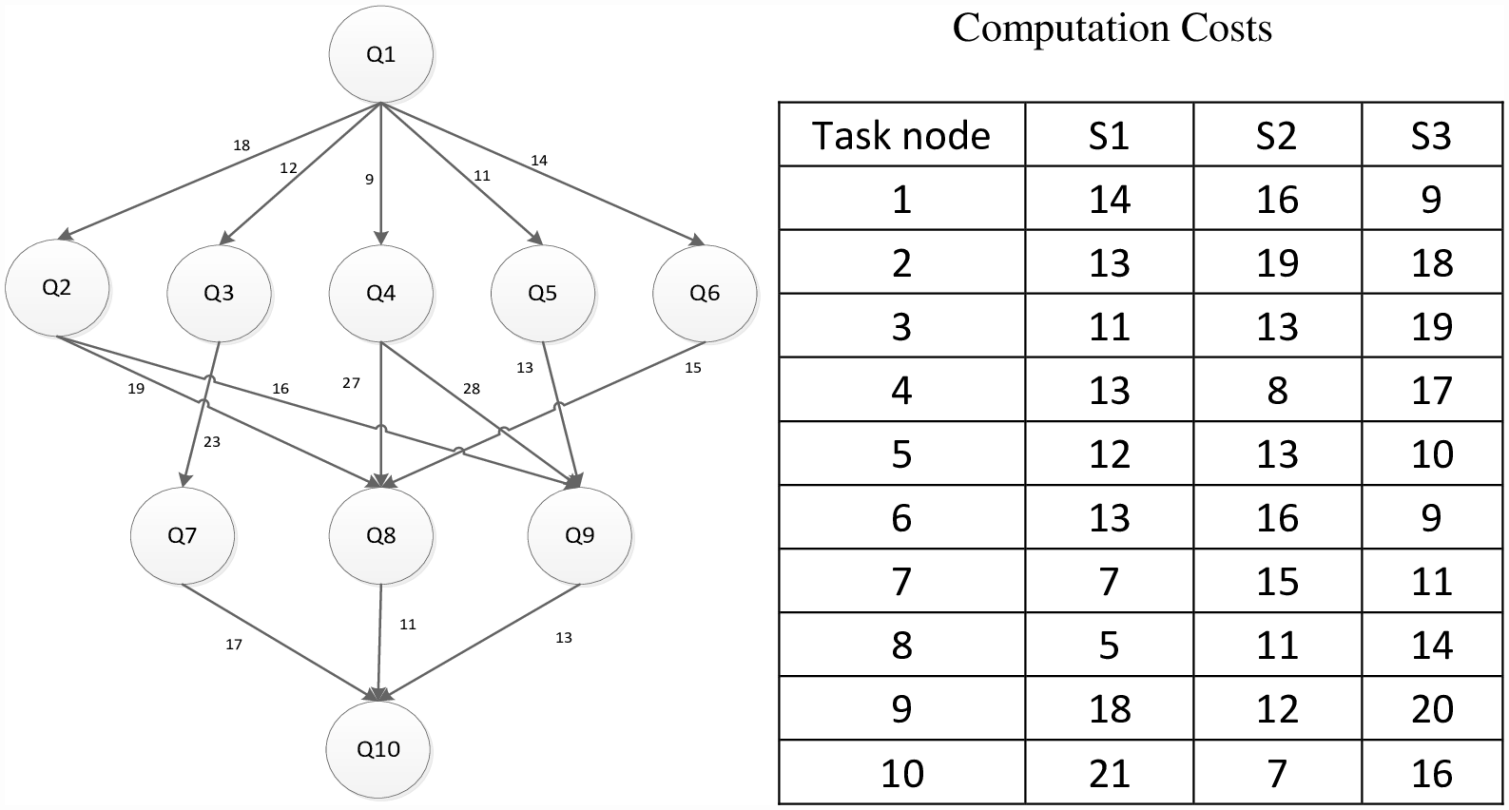
DAG Graph and the computation cost of each task node on all servers.

This paper converts an application into a DAG graph shown in Fig 6 (left). The computation costs on the three different (heterogeneous) servers are also given in Fig 6 (right). It is assumed that three servers are connected with communication links of the same capacity. Thus, the communication cost between task nodes is determined by the edge of the DAG graph shown in Fig 6 (left). We demonstrate the implementation process of DDEP algorithm and illustrated a comparative analysis between DDEP algorithm and HEFT, HEFT-Lookahead, CEFT algorithms.

DDEP algorithm is a scheduling algorithm for an application (task) in the cloud computing system and, contains four major data phases: The computation cost phase of all task nodes on all servers, the communication cost phase between task nodes; the task node list phase with different priority values, the dynamic essential path phase of all task nodes.

(1) Computation cost phase and communication cost phase

The two phases are an original array. The user application (task) owns the phase or table that stores the computation cost of each task node on the different servers. The communication cost between task nodes is stored by the adjacent matrix.

(2) Task node list with different priority values

Each task node in the Fig 6 is assigned a priority value by the predecessor-task layer priority strategy. According to the different priority values, all task nodes are divided into groups, which are stored by linked lists, as shown in the Table 1.

**Table 1:** Task nodes by grouping.

Priority value	Task node list
4	Q1
3	Q2, Q3, Q4, Q5, Q6
2	Q2, Q3, Q4, Q5, Q6
1	Q10

(3) Dynamic essential path (DEP) phase

**Fig 7:**
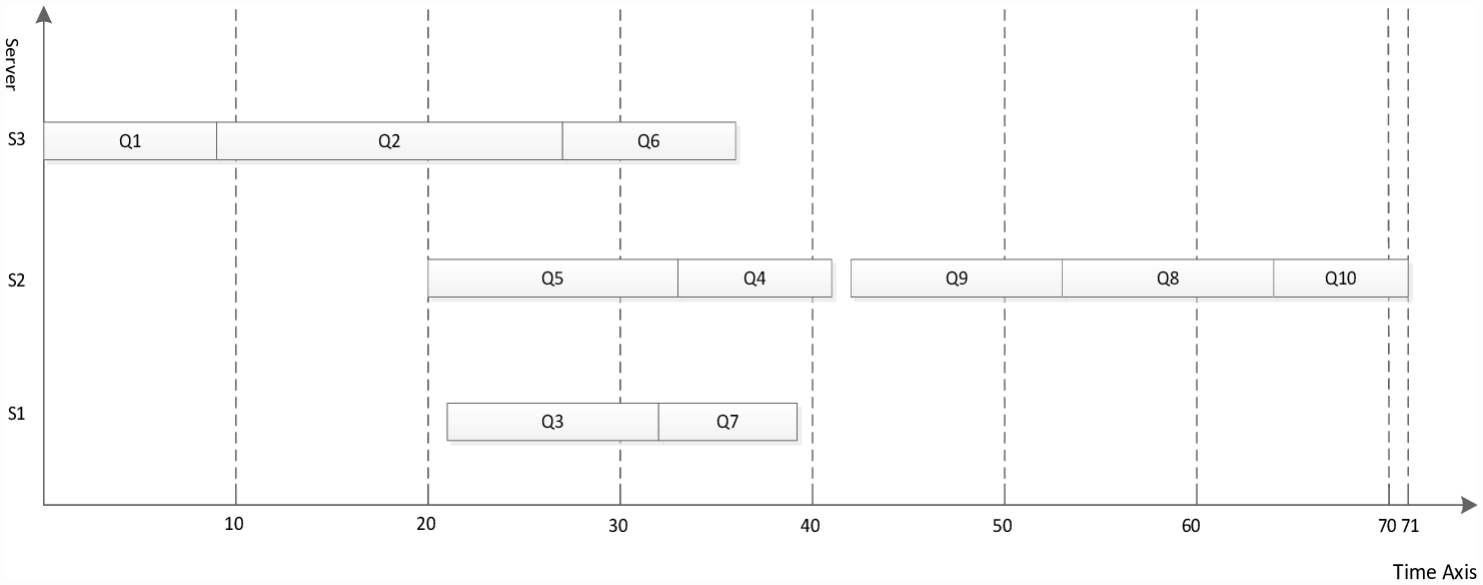
Scheduling of the DAG graph in Fig 6 using the proposed algorithm.

The dynamic essential path of all task nodes is initialized as 0. The task node list of highest priority value is sought from the task node list, and the entry task node is found, Task_node_4 = Q1. The DEP of Q1 is abtained by formulas (7) and (8), DEP_Q1 = 13. Because the task node with the priority value of 4 is the only one, the task node order is not sorted. The S3 that minimizes Q1 finish time is selected to schedule according to the computation cost phase and communication cost phase. The processing server of Q1 to S3 is updated, the computation cost is set to 9, and DEP_Q1 is set to 9. The task node with a priority value of 3 is searched, Task_node_3 = {Q2, Q3, Q4, Q5, Q6}. The DEP values of Q2, Q3, Q4, Q5, Q6 are updated to {27,21,18,20,23}. Because the task node with a priority value of 3 is not the only one, the task nodes order are sorted by the DEP value in descending order. The scheduling order is Q2, Q6, Q3, Q5 and Q4. The corresponding server is selected that minimizes each task node finish time for scheduling one by one manner. After the task nodes Q2, Q6, Q3, Q5 and Q4 are completely scheduled, the computation cost phase and communication cost phase are updated and the DEP of task nodes Q2, Q6, Q3, Q5 and Q4 to 45, 32, 26, 33 and 32 respectively. The scheduling result using the proposed algorithm is shown in Fig 7.

**Fig 8:**
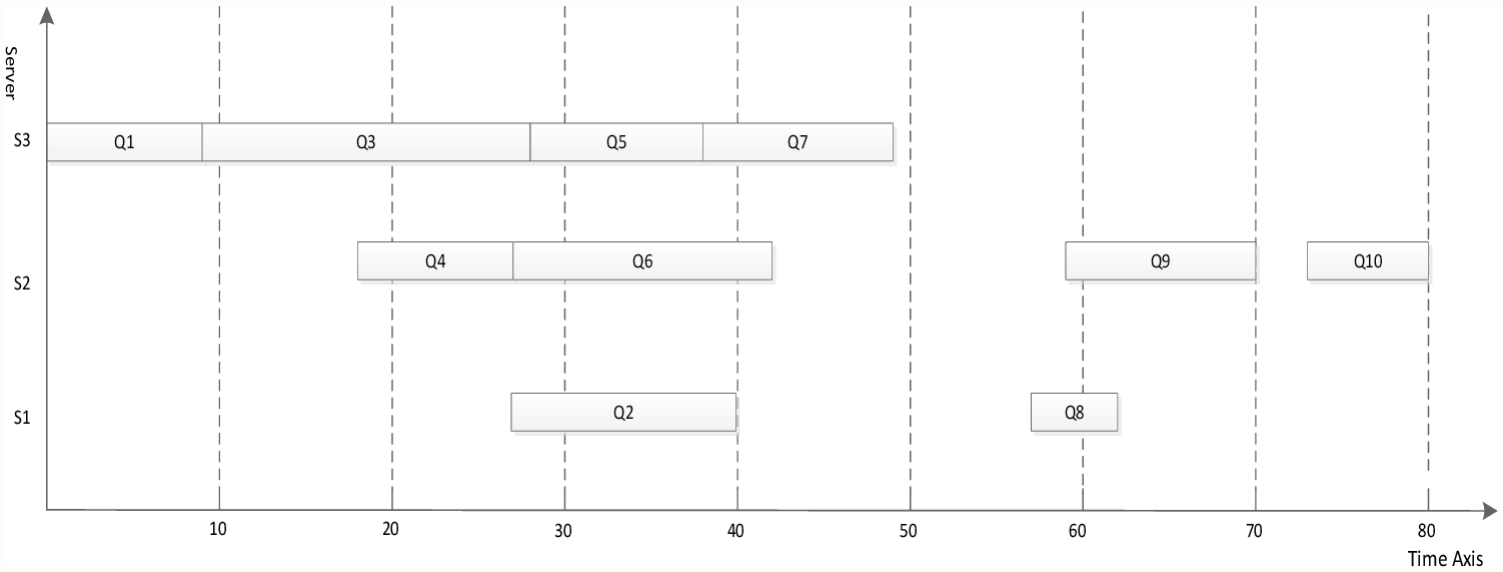
Scheduling of the DAG graph in Fig 6 using HEFT algorithm.

**Fig 9:**
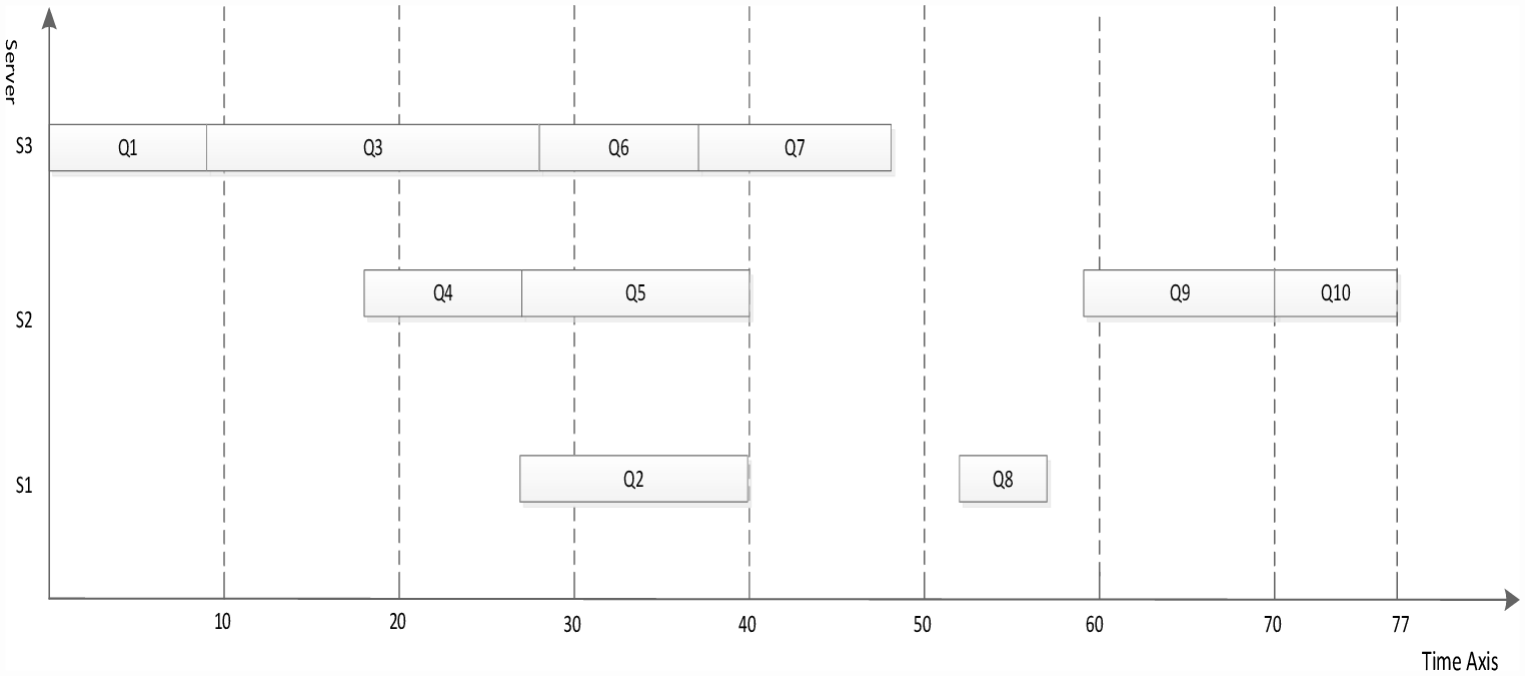
Scheduling of the DAG graph in Fig 6 using HEFT-Lookahead algorithm.

**Fig 10:**
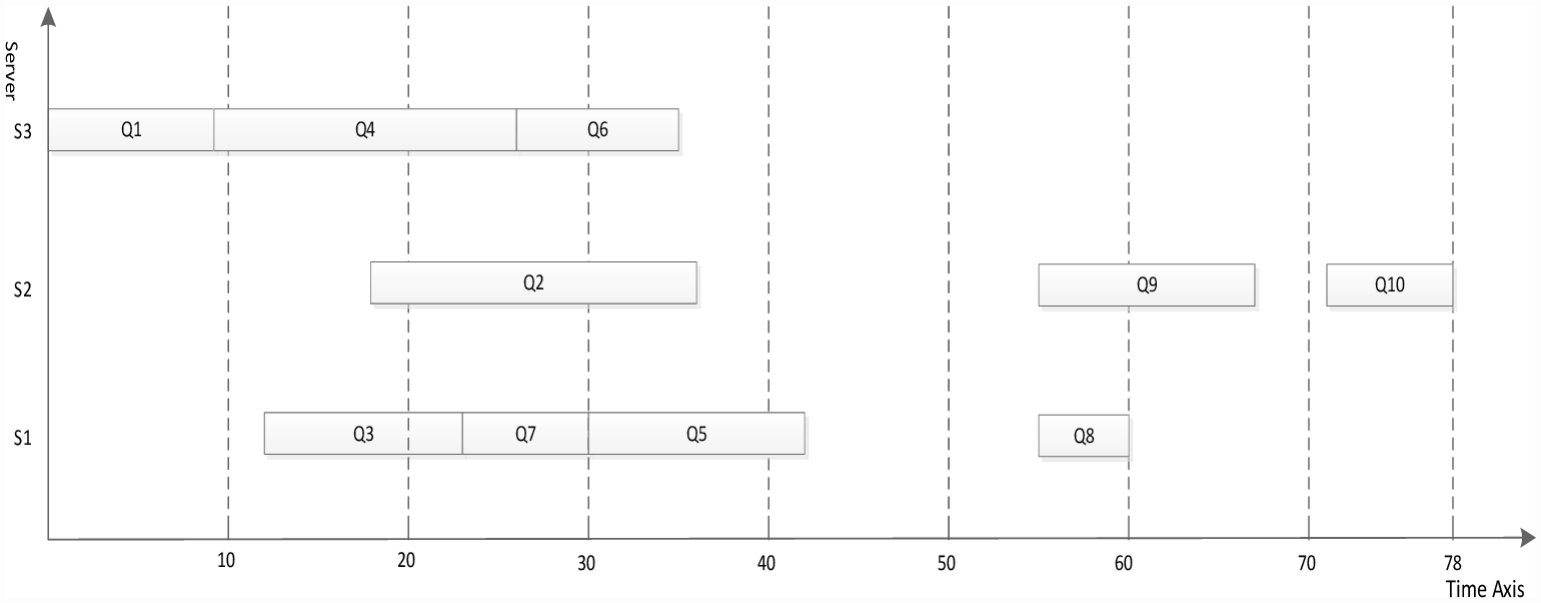
Scheduling of the DAG graph in Fig 6 using CEFT algorithm.

Figs 7–10 display the scheduling result for Fig 6 using DDEP, HEFT, HEFT-Lookahead and CEFT algorithms respectively. The scheduling result from the proposed algorithm is the shortest compared with those of HEFT, HEFT-Lookahead and CEFT algorithms. Because the proposed algorithm fully considers the influence of the scheduling order problem according to the priority value and dynamic essential path of task node.The task node that has the higher priority value and longer dynamic essential path is scheduled first, which shortens the completion time of task graph. According to scheduling result contrast analysis, the completion time of DDEP algorithm is better than those of HEFT algorithm, HEFT-Lookahead algorithm and CEFT algorithm by 12.7%, 10% and 8.5%, respectively. For example, for task node Q2, if considering the priority value and the dynamic essential path, the Q2 is scheduled first, which shortens the communication cost between Q2 and Q1. The communication cost between Q2 and Q1 is greater compared with the communication cost between the same priority value task nodes and Q1. If Q2 is not scheduled first, the start time of the successor task nodes of Q2 is inevitably influenced by the communication cost between Q2 and Q1, and in this manner, the completion time of task graph is influenced.

## Complexity Analysis

The time complexity of DDEP algorithm contains two separate components: one is the time complexity, for which the priority value of all task nodes is obtained by the predecessor-task layer priority strategy, and the other is the time complexity, for which the dynamic essential path of all task nodes is obtained by the dynamic essential long path strategy. It is assumed that *n* is the number of task nodes, and *k* is the number of servers. The specific time complexity analysis is described as follows.

1. The time complexity of the predecessor-task layer priority strategy. The adjacent matrix is used to store the relationships(communication cost) between task nodes in the task graph. The number of task nodes is *n*, and the size of adjacent matrix is *n* * *n*. The time complexity of searching for the exit task node is *O*(*n*). Then search for the predecessor task nodes of the exit task node, the column data of the exit task nodes are traversed, and the maximum number of comparisons is *n*. The priority value of each task node is obtained in the lookup procedure. Subsequently, the maximum comparison time that the priority value of all task nodes obtains is *n* * (*n* − 1), i.e., *O*(*n*^2^). To sum up, the time complexity of the predecessor-task layer priority strategy is *O*(*n*^2^)+*O*(*n*).
2. The time complexity of the dynamic essential long path strategy. First the maximum number that the highest priority value of task node can be obtained is (*n* − 1), and the number of computations of the dynamic essential path of these task nodes is (*n* − 1). Second, the process selects the corresponding server for each task node according to the sort order of the dynamic essential path, with counting time *k* * (*n* − 1). The dynamic essential path of each task node is updated, and the counting time is (*n* − 1). Thus the dynamic essential path of the same priority task node is obtained to require a counting time of 2 * (*n* − 1) + *k* * (*n* − 1). It is assumed that the highest priority value is *h*. The dynamic essential path of all task nodes is obtained to require the counting times for *h* * (2 * (*n* − 1) + *k* * (*n* − 1)) = (2 + *k*)(*n* * *k* * *h* − *h*). In the worst case, *h* = *n*, because the number of servers is relatively smaller than the number of task nodes, and the time complexity of computing the dynamic essential path is *O*(*n*^2^).
3. The time complexity of sorting all task nodes by the dynamic essential path. All task nodes are sorted in descending order by the dynamic essential path, whose time complexity is *O*(*n* log *n*).

To summarize, the time complexity of DDEP algorithm is *O*(*n*^2^)+*O*(*n* log *n*)+*O*(*n*^2^)+*O*(*n*), which is approximated as *O*(*n*^2^). The time complexities of the algorithms in references [20–22, 24] are *O*(*n*^3^), *O*(*n*^3^), *O*(*n*^3^), and *O*(*n*^2^ log *n*), respectively, which are higher than the time complexity of DDEP algorithm.

## Experiment Result and Comparison

In this section, we present simulation experiments on the DDEP algorithm. The experimental model is a rather typical computing model—DAG scheduling model. The simulation experiments are organized as follows. In the first section, the experiment environment is introduced. In section 2, the performance results are presented, and the experimental results for the algorithm execution times are covered in section 3.

### Experimental environment

We choose MATLAB (The Math Works Company, United States) for the simulation experiments and use HEFT algorithm, HEFT-Lookahead algorithm and CEFT algorithms in benchmark experiments to obtain a relatively objective evaluation. We designed a random generator to ensure the accuracy of the simulation experiments. The generator depended on several input parameters according to user requirements. The corresponding input parameters are listed as follows:

1. n: Number of task nodes in the graph.
2. m: Number of servers in the graph.
3. [Min_Computation_Cost, Max_Computation_Cost]: Computation time is randomly generated in the range. Max_Computation_Cost is the maximum computation time, Min_Computation_Cost is the minimum computation time.
4. MaxOutDegree: Maximum out-degree of task node. The out-degree range of the non-exit task node is 1 to MaxOutDegree. For the exit task node, the out-degree is 0.
5. MaxInDegree: Maximum in-degree of task node. The in-degree range of the non-exit task node is 1 to MaxInDegree. For the entry task node, the in-degree is 0.
6. [Min_Communication_Cost, Max_Communication_Cost]: Communication cost is randomly generated in the range.

The following experiment results are acquired, as generated with scheduling of the randomly generated DAG graph using HEFT, HEFT-Lookahead and CEFT algorithms.

### Performance Metrics analysis

To evaluate the performance of the proposed algorithm, we adopt the common performance comparison metrics *SLR*(*Schedule* *length* *ratio*) and Speedup.

SLR is the main performance measure for a scheduling algorithm on a graph, and is the ratio of the total processing time (*Makespan*) to the critical path length with a formula defined by:

$$SLR=\frac{Makspan}{\sum_{Q_{i}\in CP_{\text{min}}}\underset{S_{j}\in S}{\text{min}}\left\{T_{i}{}_{j}\right\}}$$ (12)

where *CP*_*min*_ is the set of the task nodes on the critical path of DAG graph, and the denominator is the summation of the minimum computation costs of all task nodes on the critical path. The SLR value is not less than 1. If the SLR value is smaller, the algorithm performance is better; if the algorithm performance is worse, the SLR value is larger. The average SLR values over several DAG graphs are used in our experiment.

Speedup is the ratio of the sequential execution time to the total processing time (Makespan). The speedup formula is defined by:

$$Speedup\;=\;\frac{\underset{S_{j}\in S}{\text{min}}\left\{\sum_{T_{i}\in V}T_{i}{}_{j}\right\}}{Makspan}$$ (13)

The sequential execution time is equal to the summation of the computation costs of all task nodes on the single server that minimizes the computation cost. If the Speedup value is smaller, the algorithm performance is worse, whereas if the speedup value is larger, the algorithm performance is better. The average Speedup values over several DAG graphs are used in our experiment.

**Fig 11:**
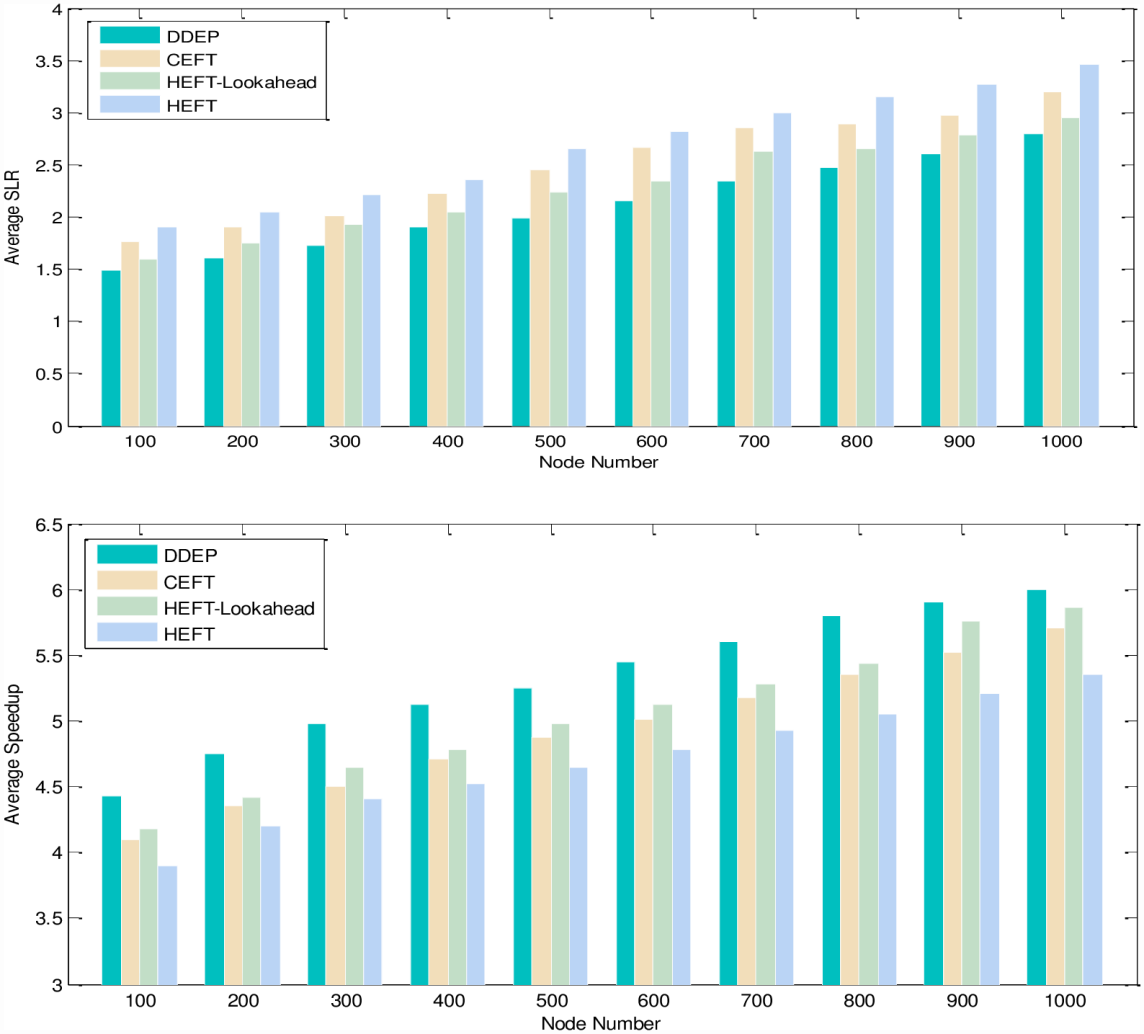
Average SLR and Average Speedup with respect to DAG graph size.

**Fig 12:**
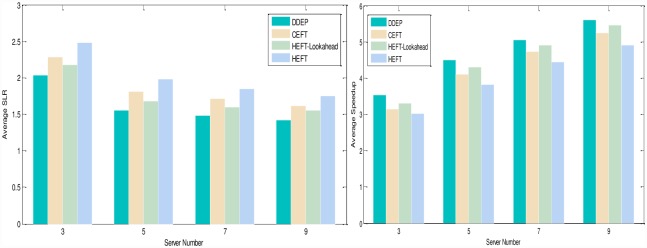
Average SLR and average Speedup with respect to server number.

**Fig 13:**
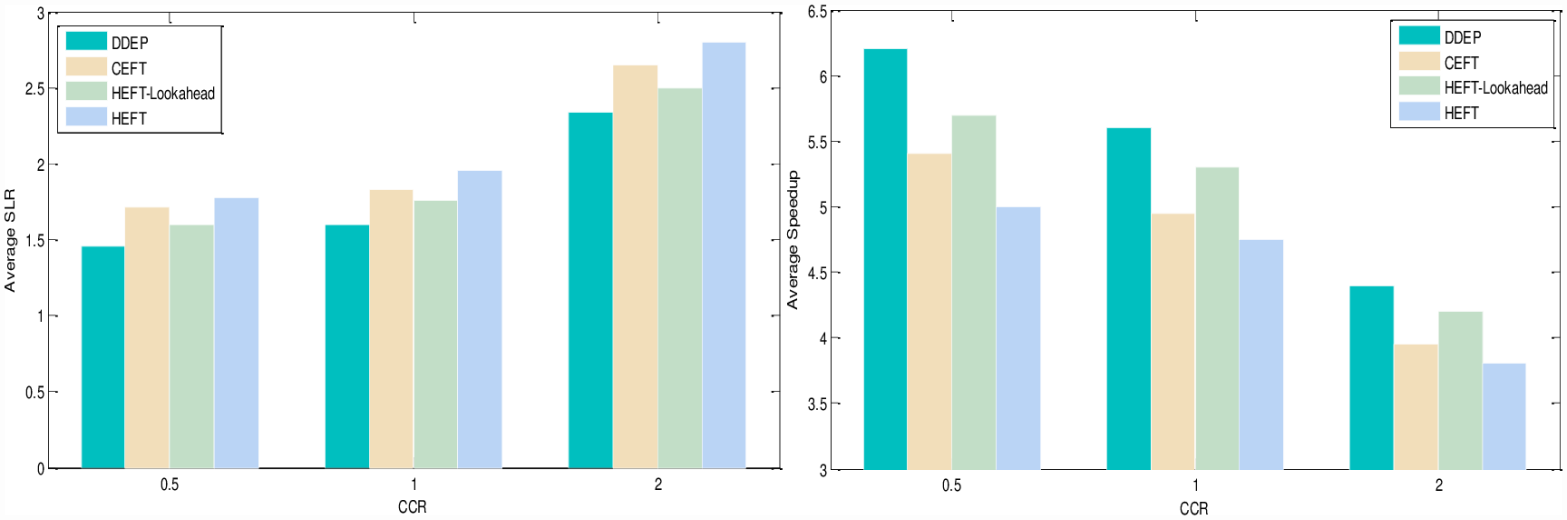
Average SLR and average Speedup with different CCR values.

**Fig 14:**
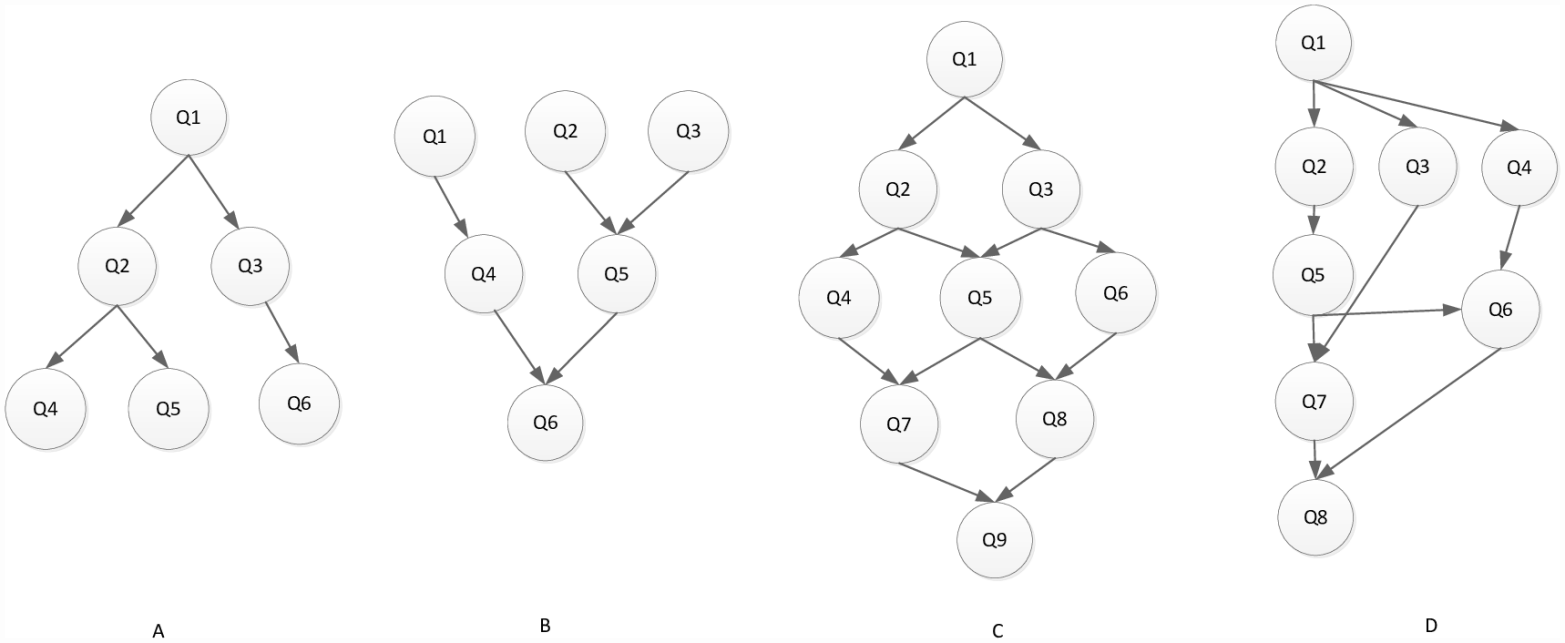
Different type DAG Graph: A: an out-tree task graph, B: an in-tree task graph, C: a mean value analysis task graph, D: an LU-decomposition task graph.

**Fig 15:**
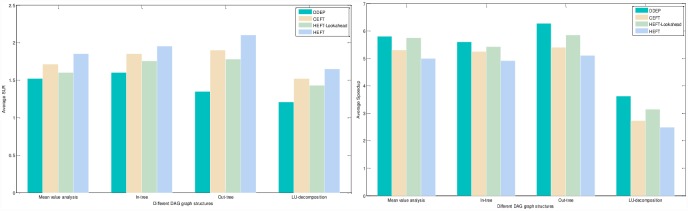
Average SLR and average Speedup with different DAG graph structures.

1. Experimental analysis of the task graph scale. The goal is to verify the influence of the task graph scale on the scheduling algorithm. To show the performance of the proposed algorithm, we adopt different sizes of DAG graph that are scheduled on the same size server to obtain the experimental result. We set the number of task nodes as 100, 200, 300, 400, 500, 600, 700, 800, 900, 1000 respectively, the number of servers is also 5. The computation cost is generated randomly in the interval 5–10, and the communication cost is generated randomly in the interval 5–10. The out-degree and in-degree of task graph are also randomly generated in the interval 1-10. Fig 11 shows the obtained comparative results for average SLR and average Speedup, as averaged over 100 runs for the same scale task graph. According to the contrast analysis of the experimental result, the average SLR of DDEP algorithm is better than those of HEFT algorithm, HEFT-Lookahead algorithm and CEFT algorithm by 25.5%, 11.3% and 16.1% respectively. The average Speedup of DDEP algorithm is better than those of HEFT algorithm, HEFT-Lookahead algorithm and CEFT algorithm by 17.8%, 10.1% and 14.1% respectively.
2. Experimental analysis of the server scale. The goal is to verify the influence of the server scale on the scheduling algorithm. We adopt the same size DAG graph and schedule it on the different sizes of server to obtain the experiment result. We set the number of task nodes to 100, and the number of servers to 3,5,7 and 9. The computation cost is randomly generated from the interval [5, 10]. The communication cost is randomly generated from the interval [5, 10]. The out-degree and in-degree of task graph are also randomly generated from the interval [1, 10]. Fig 12 shows the obtained comparative results for the average SLR and average Speedup, as averaged over 100 runs on the same scale server. according to the contrast analysis of the experimental results, the average SLR of DDEP algorithm is better than those of HEFT algorithm, HEFT-Lookahead algorithm and CEFT algorithm by 23.5%, 10.8% and 12.1% respectively. The average Speedup of DDEP algorithm is better than those of HEFT algorithm, HEFT-Lookahead algorithm and CEFT algorithm by 18.5%, 9.3% and 12.5%,respectively.
3. Experimental analysis of CCR (Communication Cost = CCR*Computation Cost). The goal is to verify the stability of the scheduling algorithm by converting the communication cost. The computation cost is taken from the interval [10, 20]. The communication cost is generated from the interval [5, 20]. The number of task nodes is 100. and the number of servers is 5. We create three groups of experimental data (each group includes 100 samples). Fig 13 shows the obtained comparative results for the average SLR and average Speedup values, as averaged over 100 runs. According to experimental result contrast analysis, the average SLR of DDEP algorithm is better than HEFT algorithm, HEFT-Lookahead algorithm and CEFT algorithm by 18.8%, 8.5%and 13.1%respectively. The average Speedup of the DDEP algorithm is better than those of HEFT algorithm, HEFT-Lookahead algorithm and CEFT algorithm by 17.1%, 11.2% and 15.5%, respectively.
4. Experimental analysis of robustness. The robustness of the scheduling algorithm is shown as the stability of scheduling a heterogeneous DAG graph. The computation cost is generated from the interval [10, 20], and the communication cost is generated from the interval [10, 20]. The number of task nodes is 100, and the number of servers is 3. We create three groups (each group includes 100 samples) of heterogeneous DAG graphs (as shown in Fig 14). Fig 15 shows the obtained comparative results for the average SLR and average Speedup value. According to the contrast analysis of the experimental results, the average SLR and average Speedup of DDEP algorithm are better than those of HEFT algorithm, HEFT-Lookahead algorithm and CEFT algorithm. Particularly, for the out-tree and LU-decomposition task graph, the performance of the proposed algorithm is much better.
5. Conclusion. The performance of the proposed algorithm is verified from four aspects. According to the analysis results shown in Figs 11–13 and 15, the proposed algorithm exhibits better performance than HEFT algorithm, HEFT-Lookahead algorithm and CEFT algorithm. Because the proposed algorithm fully considers the sort order of all task nodes affected by the priority value and the dynamic essential path of each task node, it makes the sort order of all task nodes more reasonable, and then this reasonable order can shorten the completion time of the task graph.

### Algorithm running time analysis

**Fig 16:**
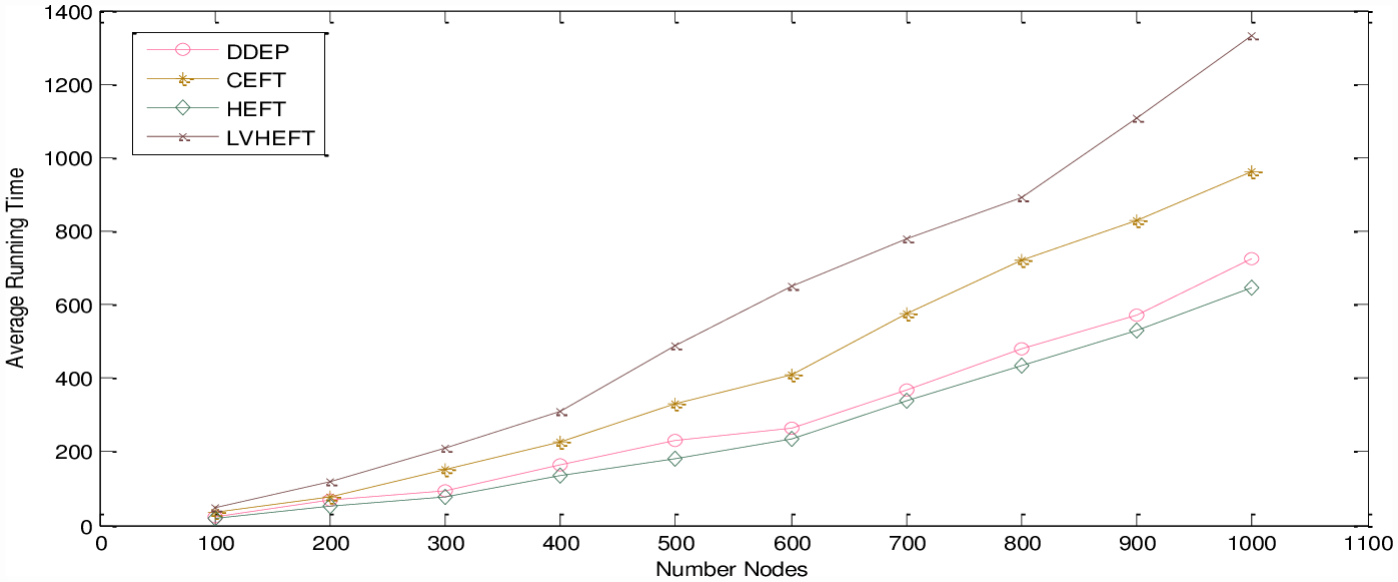
Average run time of the algorithm with respect to DAG graph size.

To evaluate the performance of the proposed algorithm, we adopt the common performance comparison metric of algorithm running time. We use the proposed algorithm to schedule different sized DAG graphs that are run 100 times on 5 servers to compute the average running time. The experimental result is shown in Fig 16. As shown in Fig 16, the running time of the proposed algorithm is longer than that of HEFT algorithm with the same size DAG graph, and much greater time is used to compute the dynamic essential path for each task node. The running time of HEFT-Lookahead and CEFT algorithms are longer than that of the proposed algorithm, and a much greater amount of time is used to search for the earliest finish time for the current task node and its immediate successor task nodes, as required for each task node by the HEFT-Lookahead algorithm. CEFT algorithm spends a greater amount time on evaluating the completion time of the sub-task graph.

Finally, for the same-size DAG graph, the running time of the proposed algorithm is longer than that of HEFT algorithm, but the completion time of the proposed algorithm is shorter than those of HEFT algorithm, HEFT-Lookahead algorithm and CEFT algorithm. Because the proposed algorithm fully considers the rationality of the priority value of each task node and because the scheduling order is affected by the change of the dynamic essential path for each task node, a greater amount time is spent on parallel scheduling. In this way, the completion time of the task graph is shortened.

## Conclusion

In this paper, we propose a scheduling algorithm for the cloud computing system based on the driver of dynamic essential path to solve the problem of the scheduling result affected by the scheduling order change of each task node in the scheduling process. Because the scheduling model is a DAG model of parallel computing, the algorithm has universality. The innovative points and significance of this paper are described as follows. The algorithm adopts the predecessor-task layer priority strategy to solve the problem in which the scheduling order of task nodes is affected by the constraint relations among task nodes. Compared with the traditional level priority, the scheduling order of task nodes is more reasonable using the proposed strategy. The algorithm uses the dynamic essential long path strategy to solve the problem in which the scheduling order of all task nodes is affected by the change of the dynamic essential path of each task node in the scheduling process. The reasonable scheduling order shortens the completion time of task graph compared with those algorithms that determine the scheduling order of task nodes prior to scheduling. The time complexity of the proposed algorithm is *O*(*n*^2^), which is lower than those of the traditional DAG scheduling algorithms. As a result, the proposed method is simple and viable. The robustness of the proposed algorithm is greater than those of the other scheduling algorithms, and thus the proposed algorithm can be used to solve the scheduling problem of the heterogeneous DAG graph. Compared with the other scheduling algorithms, the performance of the proposed algorithm is much better.

In conclusion, the proposed algorithm is able to solve the cloud computing scheduling problem and also display a certain reference value for solving the scheduling problem of parallel computing, distributed computation and grid computing.
